# Validation of reference genes for expression analysis by quantitative real-time PCR in *Leptinotarsa decemlineata* (Say)

**DOI:** 10.1186/1756-0500-6-93

**Published:** 2013-03-13

**Authors:** Xiao-Qin Shi, Wen-Chao Guo, Pin-Jun Wan, Li-Tao Zhou, Xiang-Liang Ren, Tursun Ahmat, Kai-Yun Fu, Guo-Qing Li

**Affiliations:** 1 Education Ministry Key Laboratory of Integrated Management of Crop Diseases and Pests, College of Plant Protection, Nanjing Agricultural University, Nanjing 210095, China; 2 Department of Plant Protection, Xinjiang Academy of Agricultural Sciences, Urumqi 830091, China

**Keywords:** *L. decemlineata*, Quantitative real-time PCR, Reference gene, Normalization

## Abstract

**Background:**

*L. decemlineata* is an exotic invasive insect pest, and invaded in Xinjiang Uygur autonomous region in China in the 1990s from Kazakhstan. It is a notorious defoliator of potato throughout most of the northern Xinjiang in current, and often causes extremely large yield losses of potato.

**Results:**

The expression stability of nine *L. decemlineata* house-keeping genes (*Actin*, *ACT1* and *ACT2*; *ADP-ribosylation factor*, *ARF1* and *ARF4*; *TATA box binding protein*, *TBP1* and *TBP2*; *ribosomal protein RP4* and *RP18*; *translation elongation factor 1α EF1α*) was evaluated by quantitative real-time polymerase chain reaction (qRT-PCR) in seven developmental stages, three larval tissues and two insecticide treatments. The results were analyzed using three software programs: geNorm, NormFinder and BestKeeper. Although there was no consistent ranking observed among the house-keeping genes across the samples, the overall analysis revealed that *RP18, RP4, ARF1*, and *ARF4* were the four most stable house-keeping genes. In contrast, *ACT1* and *ACT2*, two of the most widely used reference genes, had the least stability. Our results suggest that the combined use of the four most stably expressed genes may produce optimal normalization for qRT-PCR.

**Conclusions:**

The expression stability of the house-keeping genes varies among different developing stages, in different tissues and under different experimental conditions. Our results will enable a more accurate and reliable normalization of qRT-PCR data in *L. decemlineata.*

## Background

The Colorado potato beetle, *Leptinotarsa decemlineata* (Say), is an exotic invasive insect pest. It invaded in Xinjiang Uygur autonomous region in China in the 1990s from Kazakhstan. It is a notorious defoliator of potato throughout most of the northern Xinjiang in current, and often causes extremely large yield losses of potato [1-5]. *L. decemlineata* has a complicated and diverse life cycle. Moreover, the beetle has developed resistance to many classes of insecticides, among them are organophosphates, carbamates, pyrethroids and neonicotinoids [6,7].

Understanding the molecular regulation mechanisms that underlie these ecological and physiological adaptations in *L. decemlineata* may provide insights into complex regulatory networks, and may help to develop intriguing targets for the control of this pest. Using the next generation sequencing method, we have obtained a transcriptomic database. Deciphering gene expression profiles and validation of mRNA levels for target genes via quantitative real-time polymerase chain reaction (qRT-PCR) have been crucial to on-going studies. For normalizing qRT-PCR results, the mRNA levels of the target genes need to normalize to internal control house-keeping genes (HKGs) [8-10].

However, increasing evidence has suggested that HKGs varies among different insect species, in different tissue samples and under different experimental conditions [11-27]. In coleopteran species *Agrilus planipennis*, for example, *translation elongation factor 1α* (*EF1α*) was the most stable gene, whereas *glyceraldehyde-3-phosphate dehydrogenase* (*GAPDH*) and *actin* (*ACT*) showed least stability among six candidate HKGs for all the samples [12]. In another coleopteran species *Tribolium castaneum*, the most stable ones were ribosomal protein genes, *RPS3*, *RPS18*, and *RPL13a*, whereas *β-actin*, *α-tubulin*, and *RPS6* were not stable [20]. Moreover, choosing unsuitable endogenous control genes resulted in low precision or misleading results [28]. Therefore, HKGs should be validated to assure expression stability before using them as endogenous control genes in qRT-PCR.

In *L. decemlineata*, the expression stability of three HKGs, *β-actin*, *RP4* and *RP18*, was evaluated. *RP4* was the most stable among samples and was used as reference gene [29]. According to Vandesompele et al. (2002), however, at least two endogenous control genes are recommended. In the present paper, therefore, we selected 9 HKGs (*Actin*, *ACT1* and *ACT2*; *ADP-ribosylation factor*, *ARF1* and *ARF4*; *TATA box binding protein*, *TBP1* and *TBP2*; *ribosomal protein RP4* and *RP18*; *translation elongation factor 1α EF1α*) from the transcriptome and validated the stability of their expression. We have identified several suitable reference genes for gene expression studies.

## Methods

### Insect collection and rearing

Post-diapause *L. decemlineata* adults were collected from potato field in spring at Urumqi city (43.82 N, 87.61E), Xinjiang Uygur autonomous region in China. Insects were routinely reared in an insectary at 28 ± 1°C under a 14 h:10 h light–dark photoperiod and 50-60% relative humidity using fresh potato foliage as food. The adults deposited their eggs in batches of about 30 on the underside of potato leaves. After approximately 7 days, the eggs hatched into reddish-brown larvae. Larvae progressed through four distinct instars, with the average periods of the 1^st^-, 2^nd^-, 3^rd^-, and 4^th^-instar stages of 2.5, 2.5, 3.0 and 5.0 days, respectively. Upon reaching full size, the 4^th^ instars spent an additional 4–7 days as a non-feeding prepupae. The prepupae then dropped to the soil and burrowed to a depth of 3–5 cm to pupate. The pupae emerged in roughly 10 days. Both the male and female adults spent an average of 7 days to become sexually mature.

### Total RNA extraction and cDNA synthesis

*L. decemlineata* samples of developing eggs, 1^st^-, 2^nd^-, 3^rd^- and 4^th^-instar larvae, pupae (5 days after burrowing to soil), sexually mature adults (10 days after emergence) were collected from laboratory-rearing individuals. Moreover, the 4^th^-instar larvae were dissected to obtain midgut, fat body and cuticle. Furthermore, the 4th-instar larvae feeding on foliage immersed 0.09 mg/L chlorantraniliprole [1] and 0.12 mg/L spinosad [2] represented insecticide treatments. The samples were frozen with liquid nitrogen and homogenized in a tissue grinder. Then, total RNAs were extracted with TRIzol reagent (Invitrogen Co., USA) according to the manufacturer’s specifications, and were treated for 30 min at 37°C with RNase free DNase I (Ambion, Austin, TX) to eliminate traces of chromosomal DNA. The integrity of RNA was checked on a 1.5% agarose gel and visualized by ethidium bromide staining. The purity and amount of the total RNA samples were determined by NanoDrop ND-1000 spectrophotometer (Nanodrop Technologies, Rockland, DE, USA). First strand cDNA was synthesized from 1 μg total RNA using M-MLV reverse transcriptase (Takara Bio, Dalian, China) and Oligo (dT18) as the anchor primer. The reaction mixtures were incubated at 70°C for 10 min followed by 42°C for 1 h and 70°C for 15 min. The cDNA samples were used as templates for polymerase chain reaction (PCR).

### Selection and authentication of candidate HKGs

To search for HKG sequences from *L. decemlineata* transcriptome data, a reciprocal BLAST hits approach was used. The HKGs from other insect species in GenBank were downloaded from NCBI ( http://www.ncbi.nlm.nih.gov/), queried individually to *L. decemlineata* transcriptome using the TBLASTN program with a permissive E-value cutoff of 10^-3^ to get the hits. And then, each of the queried hits was compared back against non-redundant database of NCBI by the BLASTX program (E-value <10^-3^) to determine whether the original sequence was one of the hits. The selected HKG sequences were listed in Table 1.

**Table 1:** A list of primers of 9 candidate house-keeping genes for RT-PCR

**Gene name**^**a**^	**Primer sequences (5’to 3’)**		**Amplicon size (bp)**	**Accession no. of homolog**^**b**^
*ACT1*	Forward	GTGAGCAGTGTCCAACCTC	548	XP_966495
	Reverse	GGGAAGAGCGTAACCTTCG		
*ACT2*	Forward	GAGAAGATGGCGCAGATCATG	720	NP_001165843
	Reverse	CCACATCTGTTGGAACGTGG		
*ARF1*	Forward	GGGGAGGCAAACCGGTCA	603	XP_973025
	Reverse	GGCTTCTAAACCTAGTGCCTGG		
*ARF4*	Forward	GGACCTATCTTCAGCTATGCGT	681	XP_970752
	Reverse	CAATCCCTCGTGAAGGCCA		
*EF1α*	Forward	CCTTGTTCTGGGCAAACAGG	192	XP_968773
	Reverse	AACCTTGCCCATCAGCAC		
*RP4*	Forward	GCACCAGGTCTTGTTCGTG	311	XP_971634
	Reverse	GGGGAATACGGGCGACAG		
*RP18*	Forward	ACTTCGTGTCACTGAAACTGC	252	XP_968042
	Reverse	TATCCGCACGACTTCCTGC		
*TBP1*	Forward	ATAACCCTGGCCGTCTCCATG	886	XP_969256
	Reverse	TGTACTGTCGCCCGGGTTGAAC		
*TBP2*	Forward	TACGAGAACCCCGTACCACT	345	XP_969326
	Reverse	GCCTAACTTTGGCACCCGT		

a: ACT1 and ACT2, Actin; ARF1 and ARF4, ADP-ribosylation factor; TBP1 and TBP2, TATA box binding protein; RP4 and RP18, ribosomal protein; EF1α, translation elongation factor 1α. They are named according to the corresponding genes in the *T. castaneum* genome. b: the accession number of homologs in *T. castaneum*.

The unigenes of selected nine HKGs were assembled and clustered from short reads, there were inevitably issues with clone contamination and mix-up. Reverse transcriptase PCR (RT-PCR) was performed to authenticate the HKGs using the primers listed in Table 1. The components of PCR reaction buffer were 2.5 mM of dNTP, 10 mM of each primer, 25 mM of MgCl_2_, 5 U/μL of Ex-Taq DNA polymerase (Takara Bio, Dalian, China), in a total volume of 25 μL. Thermal cycling conditions of RT-PCR were available from the authors upon request. The amplified products were separated by electrophoresis on 1.5% agarose gel and purified using the Wizard® PCR Preps DNA Purification System (Promega). Purified DNA was ligated into the pGEM®-T easy vector (Promega) and several independent subclones were sequenced from both directions. The nucleotide sequences obtained after the sequence analysis were submitted to GenBank database (Accession No. KC190026- KC190034).

### Quantitative real-time PCR (qRT-PCR)

The qRT-PCR primers were designed using Beacon Designer 7 (Premier Biosoft International, Palo Alto, Calif., USA), and were given in Table 2. The qRT-PCR reactions were performed using SYBR Premix Ex Taq (Perfect Real Time) (Takara Co., Otsu, Japan) and ABI Real-Time 7300 PCR system (Applied Biosystems) according to the manufacturer’s protocol. The reaction mixture consisted of 2 μL of cDNA template (corresponding to 0.9 ng of the starting amount of RNA), 10 μL of SYBR Premix Ex Taq (Takara), 1 μL of forward primer (10 μM), 1 μL of reverse primer (10 μM), 0.4 μL of Rox Reference Dye (50×) in a final reaction volume of 20 μL. A reverse transcription negative control (without reverse transcriptase) and a non-template negative control were included for each primer set to confirm the absence of genomic DNA and to check for primer-dimer or contamination in the reactions, respectively. The qRT-PCR protocol included an initial step of 95°C for 30 sec, followed by 40 cycles of 95°C for 5 sec and then annealed at 60°C for 31 sec, followed by one cycle of 95°C for 15 sec, 60°C for 60 sec, and 95°C for 15 sec. PCR amplicons were subjected to melting curve analysis. The specificity of the qRT-PCR reactions was monitored with melting curve, analyzing by SDS software (version 1.4) and gel electrophoresis. Amplification efficiencies were determined by a 10-fold dilution series of template. All experiments were repeated in triplicate.

**Table 2:** Primers of 9 candidate house-keeping genes used in qRT-PCR

**Gene name**^**a**^	**Primer sequence**^**b**^	**Amplicon size (bp)**	**Slope**^**c**^	**R**^**2**^^**d**^	**Efficiency**^**e**^
*ACT1*	F-CAAAGCCAACAGGGAGAAGATGAC	105	−3.131	0.991	2.086
	R-CGACCAGAAGCGTACAAGGAGAG				
*ACT2*	F- TTCTGATTCCGTGAGGATTTTG	149	−3.313	0.985	2.004
	R- GTGAGGTGGATGTTCGTAGGG				
*ARF1*	F- CGGTGCTGGTAAAACGACAA	135	−3.131	0.964	2.086
	R- TGACCTCCCAAATCCCAAAC				
*ARF4*	F- GTGCTCGTGAACCATGTGAA	140	−3.130	0.997	2.087
	R- AACCTCCAATCCCTCGTGAA				
*EF1α*	F- CAGGGCAAGGTTTGAAAGATAA	111	−3.216	0.972	2.046
	R- CGTCTGCTTTGCGATTGAG				
*RP4*	F- AAAGAAACGAGCATTGCCCTTCCG	119	−3.317	0.984	2.002
	R- TTGTCGCTGACACTGTAGGGTTGA				
*RP18*	F- TAGAATCCTCAAAGCAGGTGGCGA	133	−3.158	0.976	2.073
	R- AGCTGGACCAAAGTGTTTCACTGC				
*TBP1*	F- ATGTCAAGCAGAAAGTCAAGAATCC	173	−3.204	0.988	2.054
	R- GCCGTAATATCCCTAACTCCCAAG				
*TBP2*	F- AGCGAGGAAGACTCCAGGTTG	171	−3.018	0.957	2.144
	R- ACTACTGAAAGAACTGTGAGTGAGC				

a: ACT1 and ACT2, Actin; ARF1 and ARF4, ADP-ribosylation factor; TBP1 and TBP2, TATA box binding protein; RP4 and RP18, ribosomal protein; EF1α, translation elongation factor 1α. b, Primer sequence of RP4 and RP18 were adopted from Zhu et al. (2011) [29]. c, Slope value of the standard curve. d, Regression coefficient calculated from the regression line of the standard curve. e, Real-time qRT-PCR efficiency calculated by the standard curve method.

### Data processing

The raw Ct values were obtained using the SDS software of ABI 7300 (version 1.4). The algorithms including geNorm [8], BestKeeper [30] and NormFinder [9] were used to analyze the stability of selected HKGs, strictly following the manuals of the algorithms.

## Results

### Selection of candidate housekeeping genes

According to the published results, nine HKGs were selected. They were given names as *ACT1*, *ACT2*, *ARF1*, *ARF4*, *EF-1α*, *RP4*, *RP18*, *TBP1* and *TBP2*, corresponding to homologous genes in the *T. castaneum* genome (Table 1). The sequence correctness of the nine HKGs was proven by RT-PCR and the obtained sequences were submitted to GenBank database.

The products from qRT–PCR were also confirmed by bi-direction sequencing. Primer specificities for qRT-PCR were verified by melting curve analysis. All primer pairs amplified a single PCR product with the expected sizes, showed a slope less than −3.0, and exhibited efficiency values ranging from 2.0–2.1 by a 10-fold dilution series of template (Table 2). These data indicate that amplification efficiencies of primers reach the standard requirements of conventional qRT-PCR.

### Expression levels of the candidate reference genes

The temporal expression patterns of mRNAs encoding the nine candidate HKGs were analyzed by qRT-PCR. All of the nine genes were expressed in seven developmental stages, and among three larval tissues, indicated by the presence of a single amplicon of the expected size on an agarose gel (data not shown).

**Figure 1:**
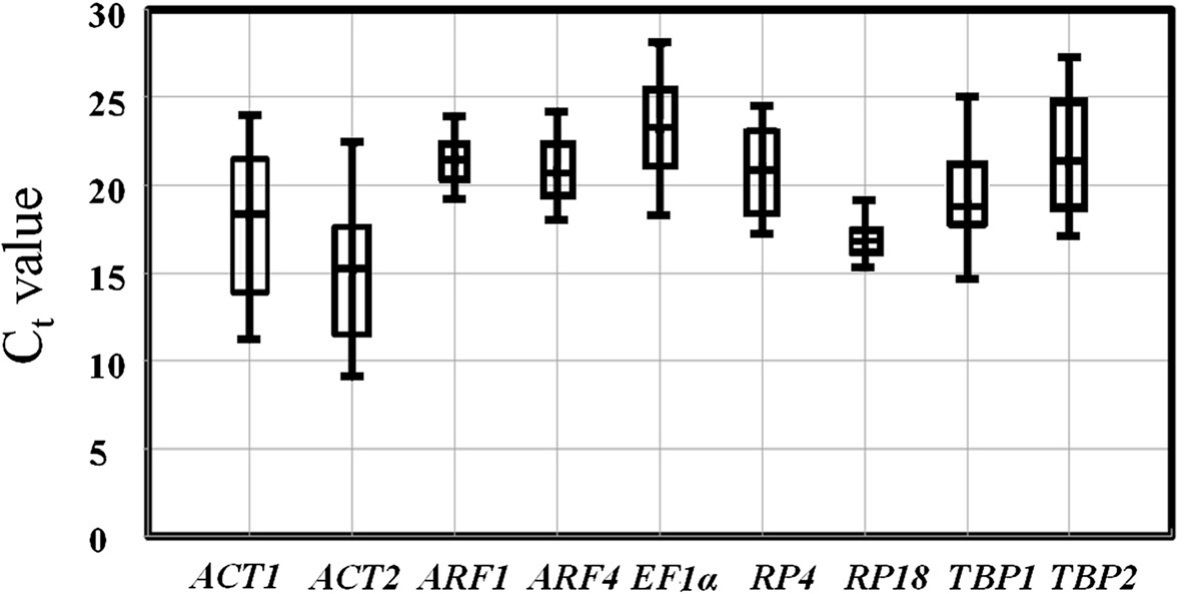
**The variation in gene expression in all tested samples of *L. decemlineata *as indicated by the raw CT values.** The box plots show the expression levels of the candidate reference genes. The values are given as the cycle threshold (CT, mean of triplicate samples). The global expression levels of the different genes analyzed are shown as the 25th and 75th quartiles (horizontal lines), median (emphasized horizontal line) and minimal to maximal value (whiskers).

Putting all the data with the same HKG at all tested samples together, the raw expression levels varied dramatically. According to the variations of Ct values, *RP18* showed the smallest gene expression variation (below ten cycles), followed by *ARF1*, *ARF4* and *RP4*, whereas *ACT2* and *ACT1* had the highest expression variation (Figure 1). Thus, determining HKGs as reference genes requires careful confirmation of expression stability.

### geNorm analysis

The geNorm defines two parameters to quantify the gene stability: M (the average expression stability) and V (the pairwise variation). The gene with the lowest M value is considered to have the most stable expression, while the one with the highest M value has the least stable expression. The V value should be below the default cut-off value of 0.15.

**Figure 2:**
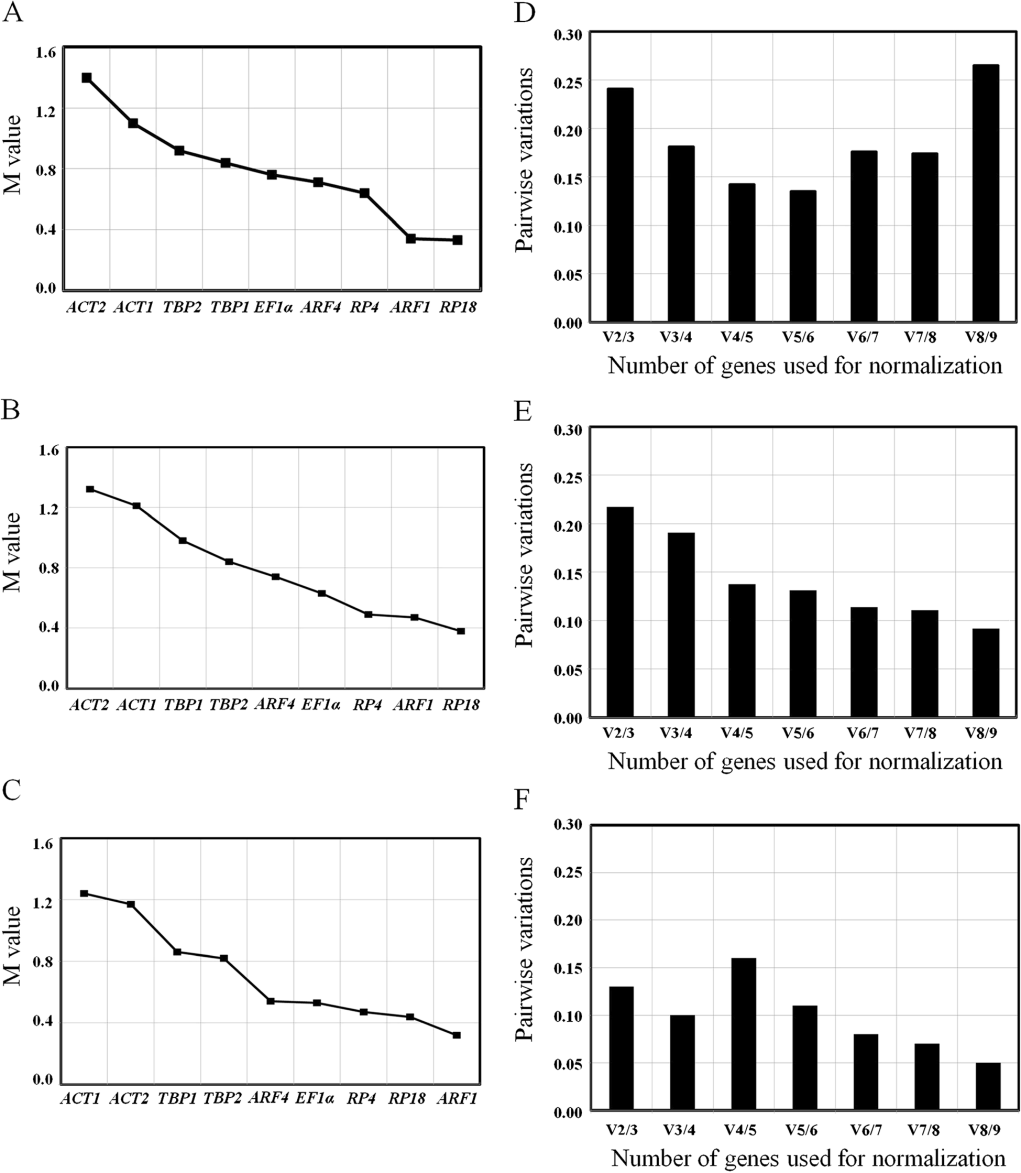
**Ranking, stability and determination of optimal number of reference genes using geNorm. A**, **B** and **C** represent ranking based on average expression stability value ( M) for developmental stages, tissues and treatments. In the plots, genes were ordered from least (left) to most (right) stable. **D**, **E** and **F** display optimal number of genes required for the accurate estimation of the target gene mRNA in developmental stages, tissues and treatments calculated by the pairwise variation (V) of the normalization factors (NFn and NFn + 1). A large pairwise variation indicates that the added gene has a significant effect and should preferably be included in the normalization. The cut-off value for such significance should be 0.15.

For developmental stages, *RP18* and *ARF1* were the best reference genes, with M-values below 0.5. *RP4*, *ARF4*, *EF1α*, *TBP1* and *TBP2* followed, that showed M-values between 0.5 and 1.0. *ACT1* and *ACT2* were the least stably expressed genes, with the M-values more than 1.0 (Figure 2A). The V2/3 and V3/4 values (the pairwise variation when the number of normalization factors is increased from two to three and from three to four) were above 0.15, whereas the V4/5 value was below 0.15 (Figure 2D). For different tissues, the stabilities of selected HKGs were *RP18* > *ARF1* > *RP4* > *EF1α* > *ARF4* > *TBP1* > *TBP2* > *ACT1* > *ACT2* (Figure 2B). The V2/3 and V3/4 values were above 0.15, whereas the V4/5, V5/6, V6/7, V7/8 and V8/9 values were below 0.15 (Figure 2E). For different treatments, the stabilities of selected HKGs were *ARF1* > *RP18* > *RP4* > *EF1α* > *ARF4* > *TBP2* > *TBP1* > *ACT2* > *ACT1* (Figure 2C). The V2/3, V3/4, V5/6, V6/7, V7/8 and V8/9 values were below 0.15, whereas the V4/5 value was above 0.15 (Figure 2F).

In summary, geNorm analysis indicates that the combined use of the four most stably expressed genes (*ARF1*, *RP18*, *RP4* and *ARF4*) produces optimal normalization for qRT-PCR among different developing stages or tissues. Moreover, combining two most stable genes *ARF1* and *RP18* is sufficient to normalize the target genes among different treatments within the 4^th^-instar stage.

### NormFinder analysis

NormFinder is designed to calculate stability using the combined estimation of intra- and inter-group expression variations of the analyzed genes. According to the NormFinder, the genes that are more stably expressed are indicated by lower average expression stability values.

**Figure 3:**
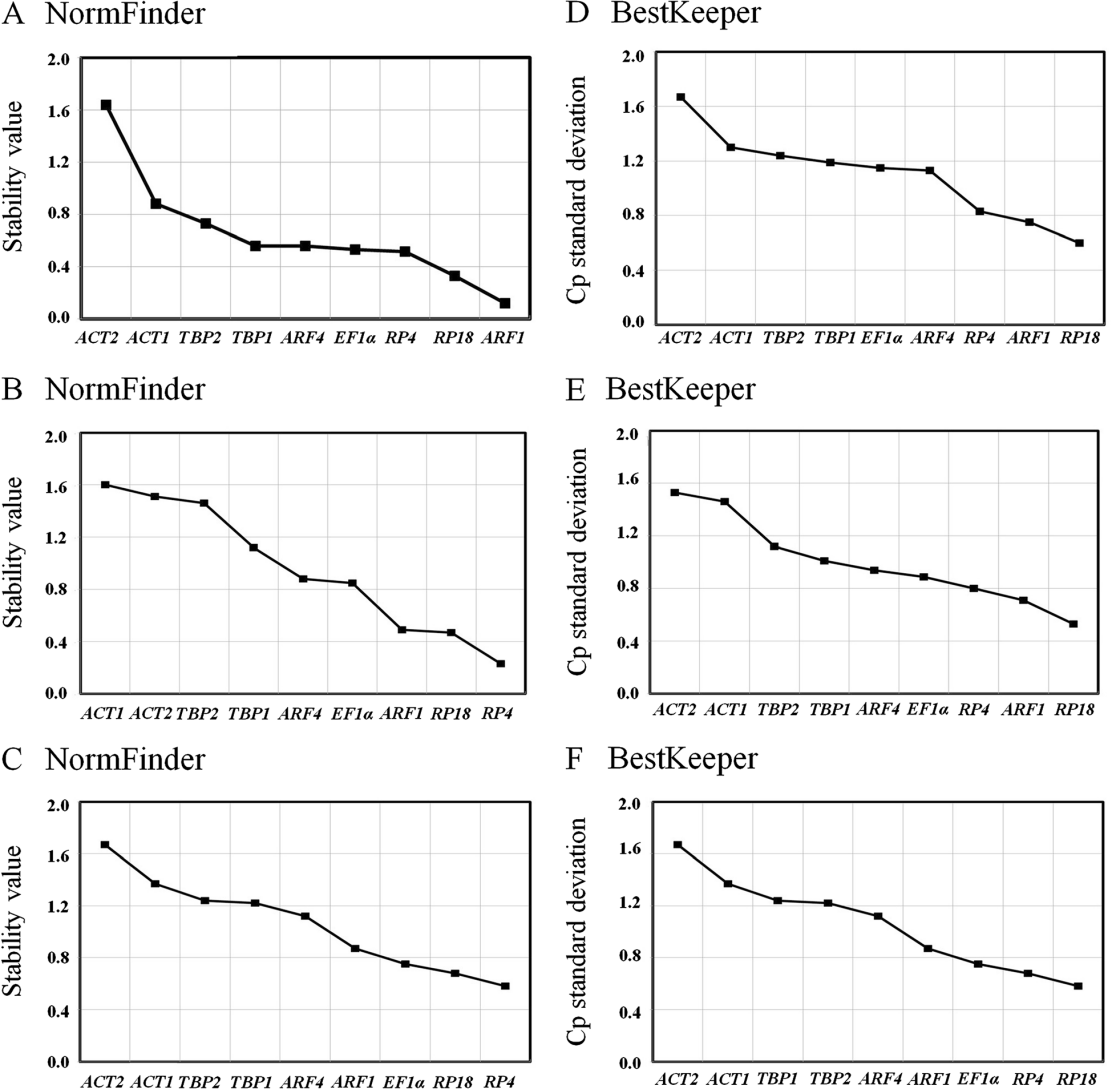
**Gene expression stability of the candidate reference genes using the NormFinder (A, B and C) and BestKeeper (D, E and F) software programs.** In the plots, genes were ordered from least (left) to most (right) stable in developmental stages (**A and D**), tissues (**B and E**) and in larvae fed on foliage immersed with different insecticides (**C and F**).

For developmental stages, the most stable genes were *ARF1* and *RP18*, the most unstable genes were *ACT1* and *ACT2* (Figure 3A). For different tissues, the stabilities of selected HKGs were *RP4* > *RP18* > *ARF1* > *EF1α* > *ARF4* > *TBP1* > *TBP2* > *ACT2* > *ACT1* (Figure 3B). Among the selected HKGs for different treatment, the four most stable genes were *RP4*, *RP18*, *EF1α* and *ARF1.* And again, the least stable genes were *ACT2* and *ACT1* (Figure 3C). Thus, the NormFinder results validate the findings of the geNorm algorithm, and *ARF1*, *RP18*, *RP4*, *EF1α* and *ARF4* are among the best combination of HKGs.

### BestKeeper analysis

According to the BestKeeper analysis, the stabilities of selected HKGs were *RP18* > *ARF1* > *RP4* > *ARF4* > *EF1α* > *TBP1* > *TBP2* > *ACT1* > *ACT2* in developmental stages (Figure 3D). The weighted index BestKeeper calculated for the nine candidates showed an SD of CP = ±0.98 cycles. The SD (±CP) value was higher for *EF1α*, *TBP1*, *TBP2*, *ACT1* and *ACT2*. This constituted a reason to exclude these genes from the BestKeeper index calculation, because they were not reliable reference genes in this setting. After the exclusion of *EF1α*, *TBP1*, *TBP2*, *ACT1* and *ACT2* from the index, its variation decreased (SD = ±0.77 cycles). After further exclusion of *ARF4* and *RP4* subsequently, the variation SD was ±0.71 and ±0.65 cycles, respectively (Table 3).

**Table 3:** Expression stability of four HKGs in different developing stages evaluated by BestKeeper software programs

**Factor**	***ARF4***	***ARF1***	***RP4***	***RP18***	**BK(n = 4)**	**BK(n = 3)**	**BK(n = 2)**
n	21	21	21	21	21	21	21
GM[CP]	23.59	21.62	19.34	16.81	20.18	19.16	19.07
AM[CP]	23.62	21.64	19.37	16.83	20.2	19.18	19.09
min [CP]	21.41	19.67	17.27	15.36	18.28	17.35	17.38
max [CP]	25.24	22.99	20.96	18.16	21.23	20.21	20.43
SD[±CP]	1.13	0.75	0.83	0.60	0.77	0.71	0.65
CV[%CP]	4.79	3.48	4.29	3.59	3.82	3.69	3.41
min[x-fold]	−4.96	−4.2	−4.2	−2.88	3.83	3.6	3.29
max[x-fold]	3.38	2.73	3.09	2.68	2.1	2.1	2.63
SD[±x-fold]	2.25	1.72	1.82	1.54	1.73	1.65	1.59

Descriptive statistics of four HKGs based on their crossing point (CP) values. Abbreviations: n: number of samples, *GM [CP]* The geometric mean of CP, *AM [CP],* The arithmetic mean of CP, min [CP] and max *[CP]* The extreme values of CP, *SD [±CP]* The standard deviation of the *CP CV [%CP]* The coefficient of variance expressed as a percentage on the CP level, *min [x-fold] and max [x-fold]* The extreme values of expression levels expressed as an absolute x-fold over- or under-regulation coefficient, *SD [± x-fold]* Standard deviation of the absolute regulation coefficients. Results from three independent experiments are shown. In the three last columns the BestKeeper index is computed together with the same descriptive parameters, for four genes (ARF4, ARF1, RP4 and RP18) or for three genes after removal of ARF4 (ARF1, RP4 and RP18) or for two genes after removal of RP4 (ARF1 and RP18).

For different tissues, *RP18*, *ARF1* and *RP4* were the most stable because they showed SD values lower than 1. *EF1α*, *ARF4*, *TBP1* and *TBP2* had SD values around 1.2. And *ACT1* and *ACT2* were the most unstable genes (Figure 3E). Among the selected HKGs for different treatment, the four most stable genes were *RP4*, *RP18*, *EF1α* and *ARF1* due to their SD values lower than 1. And again, the least stable genes were *ACT2* and *ACT1*, with their SD values more than 1.2 (Figure 3F).

In summary, BestKeeper analysis validates the findings of the geNorm and NormFinder algorithms.

## Discussion

Detection and quantification of transcript abundance in different conditions are important tasks in molecular biology. qRT-PCR is an exceptional and trustworthy technique even for low abundant mRNA transcripts [31]. However, the variations of qRT-PCR will be unavoidably introduced during RNA preparation, cDNA synthesis, and PCR process. The use of HKGs as reference genes to normalize gene expression is a strategy to minimize the variations of qRT-PCR. A good reference gene should meet three criteria. Firstly, its amplification efficiency is similar to the target genes. Secondly, it is expressed at moderate level. Lastly, its expression is stable in all test samples [11]. Unfortunately, almost all HKGs are regulated by other ‘regulators’, no gene is constitutively expressed in all cell types and under all experimental conditions [32]. Therefore, HKGs should be validated before using them as reference genes.

Evaluation of the expression stability of HKGs requires mathematical methods. Many algorithms such as geNorm [8], NormFinder [9], BestKeeper [30], ΔCt approach [33], and stability index [34] have been developed. Among them geNorm, BestKeeper and NormFinder are the most common statistical algorithms. Moreover, the three algorithms are easy to use and freely available for download [28,30,35-38]. geNorm and NormFinder use different mathematical methods to estimate the expression stability. The results of two algorithms can be used for cross validation [8,9]. Thus, we used geNorm, NormFinder and BestKeeper to identify suitable reference genes for gene expression studies in the present paper.

Ribosomal proteins are involved in translation and protein synthesis. In this study, we found that *ribosomal protein RP4* and *RP18* were among the most stable reference genes in *L. decemlineata*. Consistent with our results, ribosomal proteins are reported to be the best reference genes in many insects [22,26]. In *L. decemlineata*, *RP4* was the most stable among three HKGs, and was used as control gene [29]. In other coleopterans, *RPS3*, *RPS18*, and *RPL13a* were suitable reference genes in *T. castaneum*[20], and *RPL7* was the most stable in *A. planipennis*[12]. In lepidopterans, *RP49* had the greatest stability in different tissues of *Chilo suppressalis*, *Bombyx mori* and *Spodoptera exigua*[11]. In dipteran *Drosophila melanogaster*, *L32* was the most stable across three different treatments (i.e. injury, heat-shock stress and diets) [15]. In hemipterans, *L26*, *RPS18, RPL18* and *RPS9* were suitable reference genes in *Rhodnius prolixus*[13], *Delphacodes kuscheli*[16], *Cimex lectularius*[17] and *Aphis glycines*[14], respectively.

ADP-ribosylation factors (ARFs) are ubiquitous in eukaryotic cells. ARFs are soluble, or associate with membranes due to their N-terminus myristoylation. They function as regulators of vesicular traffic and actin remodeling [39]. Previous results revealed that *ARF* was one of the most stable HKGs in plants such as wheat, barley, rye and citrus [40-42]. In the present paper, we provided the first line of evidence that *ARF1* and *ARF4* are also stably expressed in animal species such as *L. decemlineata*.

*EF1α* encodes a protein that is involved in protein synthesis. It is widely used as reference gene in gene expression studies in plants and animals, including insects [12,21,23]. Our data showed that *EF1α* exhibited moderate stability across the samples assayed in *L. decemlineata*. In contrast, *EF1α* was the most appropriate reference gene in another coleopteran *A. planipennis*[12], in dipteran *D. melanogaster*[15], in hemipteran *A. glycines*[14], in orthopteran *Schistocerca gregaria*[23] and *Chortoicetes terminifera*[18], and in hymenopteran *Bombus terrestris* and *Bombus lucorum*[22]. Meanwhile, *E2F* was the most stable gene in different tissues of lepidopteran *B. mori*, *S. exigua* and *C. suppressalis*[11].

The TATA-binding protein (TBP) is a general transcription factor that binds specifically to a DNA sequence called the TATA box. Our data showed that *TBP1* and *TBP2* were among the least stable across the samples in *L. decemlineata*. In contrast, *TBP* showed high stability across the samples assayed in *A. glycines*[14].

Actins play key roles in cell motility and cytoskeleton maintenance. Therefore, they are assumed to be constitutively expressed and widely used as reference genes. Actin was among the most stable in collembolan *Folsomia candida* and *Orchesella cincta*[24], orthopteran *S. gregaria*[23], hemipteran *R. prolixus*[13] and *D. kuscheli*[16], siphonapteran *Lepeophtheirus salmonis*[43], dipteran *D. melanogaster*[15] and hymenopteran *Apis mellifera*[26]. However, actin genes have been challenged for their suitability as the internal controls [44]. In lepidopteran insects, *actin A1* was the most stable in *Plutella xylostella* and *C. suppressalis*, but it was the least stable in *B. mori* and *S. exigua* across different developmental stages [11]. In the present paper, we found *ACT1* and *ACT2* were the most unstable genes, irrespective of the software programs used. Similarly, *ACT* showed least stability among the candidate reference genes analyzed in *A. planipennis*[12].

## Conclusion

In summary, our work presented a number of stable HKGs that are suitable to be used as the reference genes in *L. decemlineata*. This will enable a more accurate and reliable normalization of qRT-PCR data. Moreover, these validated reference genes could also serve as the basis for selection of candidate internal controls in any given insect species.

## Abbreviations

PCR: Polymerase chain reaction; RT-PCR: Reverse transcriptase PCR; qRT-PCR: Quantitative real-time PCR; cDNA: Complementary DNA; HKG: House keeping gene; ACT: Actin; ARF: ADP-ribosylation factor; TBP: TATA box binding protein; RP: Ribosomal protein; EF: Translation elongation factor; SD: Standard deviation.

## Competing interests

The authors declare that they have no competing interests.

## Authors’ contributions

XQS and WCG performed all the experimental procedures, data analysis, and was the primary author of the manuscript. PJW, LTZ and XLR assisted in manuscript revising and provided helpful discussions. TA and KYF maintained the insects. GQL wrote the manuscript, conceived and supervised the research. All authors read and approved the final manuscript.

## References

[B1] Jiang W-H Lu W-P Guo W-C Xia Z-H Fu W-J Li G-Q Chlorantraniliprole susceptibility in *Leptinotarsa decemlineata* in the north Xinjiang Uygur autonomous region in China J Econ Entomol 2012 105 2 549 554 10.1603/EC11194 22606826

[B2] Lu W-P Shi X-Q Guo W-C Jiang W-H Xia Z-H Fu W-J Li G-Q Susceptibilities of *Leptinotarsa decemlineata* (Say) in the north xinjiang Uygur autonomous region in China to two biopesticides and three conventional insecticides J Agr Urban Entomol 2011 27 61 73

[B3] Shi X-Q Xiong M-H Jiang W-H Wang Z-T Guo W-C Xia Z-H Fu W-J Li G-Q Efficacy of endosulfan and fipronil and joint toxic action of endosulfan mixtures against *Leptinotarsa decemlineata* (Say) J Pest Sci 2012 85 519 526

[B4] Jiang W-H Guo W-C Lu W-P Shi X-Q Xiong M-H Li G-Q Target site insensitivity mutations in the AChE and LdVssc1 confer resistance 3 to pyrethroids and carbamates in *Leptinotarsa decemlineata* in northern 4 Xinjiang Uygur autonomous region Pestic Biochem Phys 2011 100 74 81 10.1016/j.pestbp.2011.02.008

[B5] Jiang W-H Wang Z-T Xiong M-H Lu W-P Liu P Guo W-C Li G-Q Insecticide resistance status of Colorado potato beetle (Coleoptera: Chrysomelidae) adults in northern Xinjiang Uygur autonomous region J Econ Entomol 2010 103 4 1365 1371 10.1603/EC10031 20857749

[B6] Alyokhin A Colorado potato beetle management on potatoes: Current challenges and future prospects Fruit, Vegetable and Cereal Science and Biotechnology 2009 3 10 19

[B7] Alyokhin A Baker M Mota-Sanchez D Dively G Grafius E Colorado potato beetle resistance to insecticides Am J Potato Res 2008 85 395 413 10.1007/s12230-008-9052-0

[B8] Vandesompele J De Preter K Pattyn F Poppe B Van Roy N De Paepe A Speleman F Accurate normalization of real-time quantitative RT-PCR data by geometric averaging of multiple internal control genes Gen Biol 2002 3 RESEARCH0034 10.1186/gb-2002-3-7-research0034 PMC126239 12184808

[B9] Andersen CL Jensen JL Orntoft TF Normalization of real-time quantitative reverse transcription-PCR data:a model-based variance estimation approach to identify genes suited for normalization, applied to bladder and colon cancer data sets Cancer Res 2004 64 5245 5250 10.1158/0008-5472.CAN-04-0496 15289330

[B10] Walker NJ A technique whose time has come Science 2002 296 557 559 10.1126/science.296.5567.557 11964485

[B11] Teng X Zhang Z He G Yang L Li F Validation of reference genes for quantitative expression analysis by real-time RT-PCR in four lepidopteran insects J Insect Sci 2012 12 60 22938136 10.1673/031.012.6001 PMC3481461

[B12] Rajarapu SP Mamidala P Mittapalli O Validation of reference genes for gene expression studies in the emerald ash borer (*Agrilus planipennis*) Insect Sci 2012 19 1 41 46 10.1111/j.1744-7917.2011.01447.x

[B13] Paim RM Pereira MH Di Ponzio R Rodrigues JO Guarneri AA Gontijo NF Araújo RN Validation of reference genes for expression analysis in the salivary gland and the intestine of *Rhodnius prolixus* (Hemiptera, Reduviidae) under different experimental conditions by quantitative real-time PCR BMC Res Notes 2012 5 1 128 10.1186/1756-0500-5-128 22395020 PMC3337225

[B14] Bansal R Mamidala P Mian MAR Mittapalli O Michel AP Validation of reference genes for gene expression studies in *Aphis glycines* (Hemiptera: Aphididae) J Econ Entomol 2012 105 4 1432 1438 10.1603/EC12095 22928326 PMC7110211

[B15] Ponton F Chapuis MP Pernice M Sword GA Simpson SJ Evaluation of potential reference genes for reverse transcription-qPCR studies of physiological responses in *Drosophila melanogaster* J Insect Physiol 2011 57 6 840 850 10.1016/j.jinsphys.2011.03.014 21435341

[B16] Maroniche G Sagadín M Mongelli V Truol G del Vas M Reference gene selection for gene expression studies using RT-qPCR in virus-infected planthoppers Virol J 2011 8 1 308 10.1186/1743-422X-8-308 21679431 PMC3142240

[B17] Mamidala P Rajarapu SP Jones SC Mittapalli O Identification and validation of reference genes for quantitative real-time polymerase chain reaction in *Cimex lectularius* J Med Entomol 2011 48 4 947 951 10.1603/ME10262 21845960

[B18] Chapuis MP Tohidi-Esfahani D Dodgson T Blondin L Ponton F Cullen D Simpson SJ Sword GA Assessment and validation of a suite of reverse transcription-quantitative PCR reference genes for analyses of density-dependent behavioural plasticity in the Australian plague locust BMC Mol Biol 2011 12 1 7 10.1186/1471-2199-12-7 21324174 PMC3048552

[B19] Shen GM Jiang HB Wang XN Wang JJ Evaluation of endogenous references for gene expression profiling in different tissues of the oriental fruit fly *Bactrocera dorsalis* (Diptera: Tephritidae) BMC Mol Biol 2010 11 1 76 10.1186/1471-2199-11-76 20923571 PMC2972281

[B20] Lord JC Hartzer K Toutges M Oppert B Evaluation of quantitative PCR reference genes for gene expression studies in *Tribolium castaneum* after fungal challenge J Microbiol Meth 2010 80 2 219 221 10.1016/j.mimet.2009.12.007 20026205

[B21] Jiang H Liu Y Tang P Zhou A Wang J Validation of endogenous reference genes for insecticide-induced and developmental expression profiling of *Liposcelis bostsrychophila* (Psocoptera: Liposcelididae) Mol Biol Rep 2010 37 1019 1029 10.1007/s11033-009-9803-0 19757170

[B22] Horňáková D Matoušková P Kindl J Valterová I Pichová I Selection of reference genes for real-time polymerase chain reaction analysis in tissues from *Bombus terrestris* and *Bombus lucorum* of different ages Anal Biochem 2010 397 1 118 120 10.1016/j.ab.2009.09.019 19751695

[B23] Van Hiel MB Van Wielendaele P Temmerman L Van Soest S Vuerinckx K Huybrechts R Broeck JV Simonet G Identification and validation of housekeeping genes in brains of the desert locust *Schistocerca gregaria* under different developmental conditions BMC Mol Biol 2009 10 1 56 10.1186/1471-2199-10-56 19508726 PMC2700112

[B24] de Boer M de Boer T Marien J Timmermans M Nota B van Straalen N Ellers J Roelofs D Reference genes for QRT-PCR tested under various stress conditions in *Folsomia candida* and *Orchesella cincta* (Insecta, Collembola) BMC Mol Biol 2009 10 54 10.1186/1471-2199-10-54 19486513 PMC2698932

[B25] Wang GH Xia QY Cheng DJ Duan J Zhao P Chen J Zhu L Reference genes identified in the silkworm *Bombyx mori* during metamorphism based on oligonucleotide microarray and confirmed by qRT-PCR Insect Sci 2008 15 405 413 10.1111/j.1744-7917.2008.00227.x

[B26] Scharlaken B de Graaf DC Goossens K Brunain M Peelman LJ Jacobs FJ Reference gene selection for insect expression studies using quantitative real-time PCR: the head of the honeybee, *Apis mellifera*, after a bacterial challenge J Insect Sci 2008 8 33

[B27] Lourenço AP Mackert A Cristino AS Simões ZLP Validation of reference genes for gene expression studies in the honey bee, *Apis mellifera*, by quantitative real-time RT-PCR Apidologie 2008 39 3 372 385 10.1051/apido:2008015

[B28] Jian B Liu B Bi Y Hou W Wu C Han T Validation of internal control for gene expression study in soybean by quantitative real-time PCR BMC Mol Biol 2008 9 59 10.1186/1471-2199-9-59 18573215 PMC2443375

[B29] Zhu F Xu J Palli R Ferguson J Palli SR Ingested RNA interference for managing the populations of the Colorado potato beetle, *Leptinotarsa decemlineata* Pest Manag Sci 2011 67 175 182 10.1002/ps.2048 21061270

[B30] Pfaffl MW Tichopad A Prgomet C Neuvians TP Determination of stable housekeeping genes, differentially regulated target genes and sample integrity: BestKeeper-Excel based tool using pair-wise correlations Biotechnol Lett 2004 26 509 515 15127793 10.1023/b:bile.0000019559.84305.47

[B31] Bustin SA Quantification of mRNA using real-time reverse transcription PCR (RT-PCR): trends and problems J Mol Endocrinol 2002 29 23 39 10.1677/jme.0.0290023 12200227

[B32] Bustin SA Absolute quantification of mRNA using real-time reverse transcription polymerase chain reaction assays J Mol Endocrinol 2000 25 169 193 10.1677/jme.0.0250169 11013345

[B33] Silver N Best S Jiang J Thein SL Selection of housekeeping genes for gene expression studies in human reticulocytes using real-time PCR BMC Mol Biol 2006 7 33 10.1186/1471-2199-7-33 17026756 PMC1609175

[B34] Brunner AM Yakovlev IA Strauss SH Validating internal controls for quantitative plant gene expression studies BMC Plant Biol 2004 4 14 10.1186/1471-2229-4-14 15317655 PMC515301

[B35] Jain M Nijhawan A Tyagi AK Khurana JP Validation of housekeeping genes as internal control for studying gene expression in rice by quantitative real-time PCR Biochem Bioph Res Co 2006 345 646 651 10.1016/j.bbrc.2006.04.140 16690022

[B36] Exposito-Rodriguez M Borges AA Borges-Perez A Perez JA Selection of internal control genes for quantitative real-time RT-PCR studies during tomato development process BMC Plant Biol 2008 8 131 10.1186/1471-2229-8-131 19102748 PMC2629474

[B37] Gutierrez L Mauriat M Guenin S Pelloux J Lefebvre JF Louvet R Rusterucci C Moritz T Guerineau F Bellini C The lack of a systematic validation of reference genes: a serious pitfall undervalued in reverse transcription-polymerase chain reaction (RT-PCR) analysis in plants Plant Biotechnol J 2008 6 609 618 10.1111/j.1467-7652.2008.00346.x 18433420

[B38] Oelkers K Goffard N Weiller GF Gresshoff PM Mathesius U Frickey T Bioinformatic analysis of the CLE signaling peptide family BMC Plant Biol 2008 8 1 10.1186/1471-2229-8-1 18171480 PMC2254619

[B39] Randazzo PA Nie Z Miura K Hsu VW Molecular aspects of the cellular activities of ADP-ribosylation factors Sci STKE 2000 http://www.stke.org/cgi/content/full/OC_sigtrans;2000/59/re1 10.1126/stke.2000.59.re1 11752622

[B40] Paolacci AR Tanzarella OA Porceddu E Ciaffi M Identification and validation of reference genes for quantitative RT-PCR normalization in wheat BMC Mol Biol 2009 10 1 11 10.1186/1471-2199-10-11 19232096 PMC2667184

[B41] Giménez MJ Pistón F Atienza SG Identification of suitable reference genes for normalization of qPCR data in comparative transcriptomics analyses in the Triticeae Planta 2011 233 1 163 173 10.1007/s00425-010-1290-y 20960006

[B42] Carvalho K de Campos M Pereira L Vieira L Reference gene selection for real-time quantitative polymerase chain reaction normalization in “Swingle” citrumelo under drought stress Anal Biochem 2010 402 2 197 199 10.1016/j.ab.2010.03.038 20363209

[B43] Frost P Nilsen F Validation of reference genes for transcription profiling in the salmon louse *Lepeophtheirus salmonis*, by quantitative real-time PCR Vet Parasitol 2003 118 169 174 10.1016/j.vetpar.2003.09.020 14651887

[B44] Selvey S Thompson EW Matthaei K LR A Irving MG Griffiths LR Beta-actin-an unsuitable internal control for RT-PCR Mol Cell Probe 2001 15 307 311 10.1006/mcpr.2001.0376 11735303

